# Investigation and analysis of magnetic resonance imaging experience and psychological status of patients

**DOI:** 10.1186/s40359-024-01570-7

**Published:** 2024-03-01

**Authors:** Shu-ping Zhou, Xin-cui Wan, Xiao-dan Wang, Xiao-man Zhang, Yun-han Yu, Wen-jun Wang

**Affiliations:** 1 Nursing Department, Hainan General Hospital, Hainan Affiliated Hospital of Hainan Medical University, Haikou, 570311 China; 2 Central Sterile Supply Department, Hainan General Hospital, Hainan Affiliated Hospital of Hainan Medical University, Haikou, 570311 China; 3 Hainan Medical University International School of Public Health and One Health, No. 3, College Road, Longhua District, Haikou, Hainan Province 571199 China; 4 Department of Comprehensive Interventional, Hainan General Hospital, Hainan Affiliated Hospital of Hainan Medical University, Haikou, 570311 China; 5 International Nursing School, Hainan Medical University, Haikou, 571199 China; 6 Office of High Quality Development Committee of the First Affiliated Hospital of Hainan Medical University, Haikou, 571000 China

**Keywords:** Magnetic resonance imaging, Nursing, Patient experience, Psychological status

## Abstract

**Objective:**

To analyze factors influencing the service experience of magnetic resonance imaging (MRI) examination and psychological status of patients admitted to a hospital and propose targeted solutions, and optimize the examination process and nursing by analyzing the MRI examination experience and psychological effect on patients.

**Methods:**

The MRI examination rooms of two tertiary general hospitals in Haikou City were sampled at random, and 206 patients who met the study criteria were surveyed on site.

**Results:**

(1) The item with the lowest mean score for patient examination services was whether earplugs were provided to the patient during the examination (B8 = 0.47). (2) Environmental logistics experience (16.83 ± 3.036) received the lowest score among the three service experience dimensions. (3) The average anxiety score of the patients was 5.38. (4) There was a positive correlation between the examination experience and the examination service experience of the patients. (5) Patients with higher monthly income had decreased anxiety (coefficient = -2.334), and MRI examination of the extremities relieved the anxiety (coefficient = -4.782).

**Conclusion:**

The environmental logistics factors, poor service attitude, examination site, and income were the most significant factors affecting the MRI examination experience and psychological status of patients, which can be improved by providing information, enhancing the waiting environment, providing targeted patient education, and evaluating the experience immediately.

**Supplementary Information:**

The online version contains supplementary material available at 10.1186/s40359-024-01570-7.

## Introduction

Magnetic Resonance Imaging (MRI) is a commonly used large-scale diagnostic apparatus in hospitals, and studies indicate that 1–30% of individuals undergoing the examination may experience severe anxiety during the process [1–3]. Anxiety related to medical procedures can result from various factors, including unfavorable expectations, unfamiliar environments, unfamiliarity with the procedure, concerns about diagnosis or prognosis, etc. [4]. Factors contributing to anxiety during MRI include the small spatial dimensions of the MRI tube, long scan times, and loud machine noises [5, 6]. Patients may also experience pre-existing discomfort, pain, uncertainty about the examination results, which can become more pronounced during extended wait times for MRI [7]. Some patients may feel tense and uncomfortable due to being confined in the narrow space of the MRI tube during the examination, leading to anxiety [8–10]. Severe anxiety reactions can disrupt ongoing cognitive or neural processes, thereby affecting MRI results and, in some cases, even triggering severe claustrophobia, making it challenging to continue or complete the examination [8, 11]. 

To alleviate anxiety and fear in patients during MRI examinations, various intervention measures have been adopted, such as audio-guided self-hypnosis, a booklet providing information about the scanning procedure and advice, using music to distract attention, and the effects of guided imagery [12–15]. These intervention measures have certain limitations, such as increased time and cost, and sometimes cognitive therapy requires specialized psychological settings. By providing information about MRI scanning procedures, instructions for the examinee, etc., through pictures, recordings, and videos, this approach significantly reduces anxiety in patients undergoing magnetic resonance imaging examinations. Through these simple modifications to the MRI imaging procedure, anxiety related to the scan can be reduced, with extremely low costs, no need for special training for staff, and no impact on the operation of the MRI imaging room [13, 16]. 

In addition to the patients’ own psychological experiences, the communication methods between staff and patients have a significant impact on patient compliance [17]. The psychological symptoms of patients undergoing MRI examinations are influenced by factors such as equipment, environment, medical personnel, and individual characteristics, leading to varying psychological states. Patient experience refers to the most direct psychological feelings of patients during their lifetime or medical treatment [18], including experiences and psychological anxiety and fear in aspects such as service level, health education, logistical support, information support, service efficiency, and emotional support. Factors such as the hospital’s overall service attitude, communication, emotional support, health education, and environment contribute to patient experience, which has become an important indicator for evaluating the quality of modern healthcare services. It is also one of the three pillars of healthcare quality, positively correlated with patient safety, clinical outcomes, and collectively impacting patient outcomes [19–21]. There is limited research on the experiences and influencing factors of patients during MRI examinations, especially regarding the overall experience of MRI examinations for inpatients.

This study aims to enhance the understanding and attention to the overall service experience and psychological symptoms of inpatients throughout the MRI examination process. By extracting and analyzing the relevant factors influencing patients undergoing MRI examinations, the goal is to effectively improve patient psychological symptoms, optimize MRI examination services, and provide a safer, more comfortable, and efficient MRI examination experience.

## Research participants and methods

### Research participants

Patients admitted to two tertiary general hospitals in Haikou City from June 1, 2021, to December 31, 2021, were recruited using a simple random sampling method. Appointment information was collected from the MRI room, and participants were selected based on inclusion and exclusion criteria. Participants who met the criteria were selected randomly by computer using their appointment number. Patient who met the following inclusion criteria were eligible for screening: (1) able to walk and communicate without barriers; (2) aged 18–65; (3) first-time examination; (4) provided informed consent and willingly participate in this study. The exclusion criteria were as follows: (1) with mental disorders or consciousness disorders; (2) with recent surgical or trauma history; (3) undergoing contrast-enhanced scans, receiving radiation therapy, lactating, or pregnant women. Finally, a total of 206 valid questionnaires were collected, for a recovery rate of 100%. There were 87 males and 119 females, for a ratio of 2:3, and the average age was 36.77 ± 10.43 years. There were 133 (64.6%) patients with fewer than two children and 73 (35.4%) with two or more children. There were 169 (82%) patients who had to support 2 or fewer elderly family members, and 37 (18%) patients who had to support 2 or more elderly family members.

### Research methods

The questionnaire used in this study was independently designed based on a review of relevant literature. General Questionnaire: There are a total of 12 items, including gender, occupation, number of children, salary, and examination location. There were 5 items for environmental logistics experience, 5 items for disease communication experience, and 5 items for service attitude experience in the Examination Service Experience Questionnaire [22, 23]. (Details are in the supplementary 1 to 3). The five-level Likert scale was used, divided into five grades of 1–5, including very satisfied (5 points), somewhat satisfied (4 points), neither satisfied nor dissatisfied (3 points), not very satisfied (2 points), and very dissatisfied (1 point). The higher the score, the higher the level of satisfaction. Symptom Check List: Two factors of anxiety and hostility were extracted for assessment from the 10 factors of the Symptom Check List (SCL90) [24, 25]. (Details are in the supplementary 4). Each item was scored on a 5-point scale: None (0 point), Mild (1 point), Moderate (2 points), Severe (3 points), and Critical (4 points). The higher the score, the greater the severity and impact.

### Reliability and validity of the questionnaires

A pre-survey was conducted on 20 eligible patients in a certain hospital to examine the questionnaire’s reliability and validity. Preliminary data analysis was performed to identify and refine influencing factors, resulting in the final questionnaire. The questionnaire’s reliability was analyzed using SPSS 25.0. In the survey on examination service experience, there were three dimensions: environmental logistics experience, disease communication experience, and service attitude experience. The overall Cronbach’s α coefficient of the questionnaire was 0.920, with a KMO value of 0.872. For the symptom self-assessment scale, which had two dimensions, anxiety and hostility, the overall Cronbach’s α coefficient was 0.934, with a KMO value of 0.916. Overall values exceeding 0.9 indicate a high level of consistency and reliability in the survey questionnaire.

### Statistical methods

Data were imported into SPSS25.0 for collection and analysis. The statistical sample size was estimated by an expert statistician based on the hypothesis of a reduction of Anxiety and hostility events from 30 to 18%. A two-tailed type 1 error of 0.05, a power of more than 80%, and an expected drop-out rate of 10% yielded a total sample size of 206 patients. Continuous variables with normal distribution were expressed as mean ± standard deviation (SD), and the Student’s t-test analyzed differences in continuous variables. Variables with non-normal distribution were presented as median (M) with the interquartile range (Q1,Q3), and the Mann-Whitney U test or Kruskal-Wallis test analyzed differences. Categorical variables were expressed as frequency (n, %). Multivariate linear regression model 1 was performed to evaluate the relationship between medical service and medical experience, linear regression model 2 was performed to evaluate the relationship between basic information, medical experience, and anxiety of inpatients; linear regression model 3 was performed to evaluate the relationship between basic information, medical experience, and hostility of inpatients. A P-value of less than 0.05 was considered to be statistically significant.

## Results

### Patient experience score and its distribution

Table 1 displays the scores for patient examination services. The lowest mean value was determined to be B8 = 0.47—“Where you provided earplugs during the examination.” B9 = 0.51—“Where you covered with a quilt during the examination,” had the second-lowest mean value. The results indicated that MRI examinations should be conducted with a heightened awareness in providing a more considerate service, such as offering earplugs to reduce examination noise and keeping patients warm during the examination.

**Table 1:** Basic situation of inpatient examination services (1 means getting the service, 0 means not getting the service)

Dimensional/secondary metrics	Mean value	Standard deviation	Minimum value	Maximum value
Examination service	6.87	1.526	2	9
B1 Hospital staff took you from the ward to the MRI room	0.76	0.427	0	1
B2 Before the examination, the ward nurse informed you of the time of examination	0.95	0.215	0	1
B3 The ward nurse educated you on the examination precautions	0.91	0.290	0	1
B4 The nurse in the MRI room educated you on the examination precautions	0.93	0.260	0	1
B5 The staff carefully checked your information before the examination	0.95	0.225	0	1
B6 The staff asked you about the examination site before the examination	0.68	0.468	0	1
B7 You were instructed on how to inform the staff if you feel unwell during the examination.	0.71	0.455	0	1
B8 You were provided earplugs for use during the examination	0.47	0.500	0	1
B9 You were covered with a quilt during the examination	0.51	0.501	0	1

The scores for environmental experience (i.e., the ambient sound of the examination room, the temperature of the examination room, and the noise generated by the equipment); disease communication experience, and service attitude experience were, 16.83 ± 3.036, 19.46 ± 2.642, and 19.99 ± 2.875, respectively, with environmental logistics experience receiving the lowest score. The score for “sound produced by the device during MRI examination” was 2.83 ± 0.785, which was the lowest of the satisfaction items, which was attributable to the equipment’s limitations and the lack of service. The scores of each item are detailed in Table 2.

**Table 2:** Scores of hospitalized patients in each dimension of medical experience

Dimensional/secondary metrics	Mean value	Standard deviation	Minimum value	Maximum value
Environmental logistics experience	16.83	3.036	6	25
C1 The ward is quiet	3.63	0.889	1	5
C2 The MRI waiting area is quiet	3.32	0.880	1	5
C3 The noise produced by the device during MRI examination	2.83	0.787	1	5
C4 The temperature of the MRI waiting area	3.50	0.744	1	5
C5 The temperature of the MRI room	3.54	0.736	1	5
Disease communication experience	19.46	2.642	5	25
C6 Patient education content at the ward	3.91	0.652	1	5
C7 Patient education content provided by the nurse in the ward	3.96	0.676	1	5
C8 Language used in the MRI room for patient education	3.76	0.689	1	5
C9 Explanation of the questions you have by the staff	3.89	0.634	1	5
C10 Your privacy is protected during the examination process	3.94	0.622	1	5
Experience of the service attitude	19.99	2.875	5	25
C11 The service attitude of the nurses in the ward	4.07	0.613	1	5
C12 The service attitude of the staff in the MRI waiting area	3.96	0.672	1	5
C13 The service attitude of technicians during MRI examination	4.01	0.613	1	5
C14 Overall satisfaction throughout the examination process	3.96	0.654	1	5
C15 Overall satisfaction of the comprehensive experience throughout the examination process	3.99	0.628	1	5
Medical experience	56.27	7.272	17	75

### Patient psychological status score and its distribution

The anxiety psychological status refers to a state characterized by the inability to remain calm, heightened nervousness, tension, and the manifestation of physical symptoms such as tremors. The mean score of the anxiety psychological status of the patients was 5.38, with the highest score (nervousness, unsteadiness) being 1.11 and the lowest score (anxiety) being 0.19 (a feeling that something familiar becomes strange or not real). The hostile psychological status refers to a state characterized by anger, tantrums, and impulsiveness. The mean hostile psychological status score was 1.11, with the highest score being 0.50 (easily annoyed and agitated) and the lowest score being 0.07 (the desire to strike or harm others) (Table 3).

**Table 3:** The basic situation of anxiety and hostility of hospitalized patients

Dimensional/secondary metrics	Mean value	Standard deviation	Minimum value	Maximum value
Anxiety	5.38	5.567	0	30
D1 Nervousness and unsteadiness	1.11	0.890	0	3
D2 Shivering	0.32	0.672	0	4
D3 Suddenly feeling scared for no reason	0.37	0.752	0	4
D4 Feeling scared	0.68	0.767	0	4
D5 Rapid heartbeat	0.65	0.806	0	4
D6 Feeling nervous or easily feeling nervous	1.04	0.814	0	4
D7 Waves of fear and panic	0.36	0.696	0	4
D8 Feeling uncomfortable and unsettled	0.35	0.643	0	4
D9 Feeling that something familiar becomes strange or not real	0.19	0.585	0	4
D10 Feel like wanting to get things done quickly	0.32	0.611	0	4
Hostility	1.11	2.483	0	17
D11 Easily annoyed and agitated	0.50	0.842	0	4
D12 Lost temper uncontrollably	0.22	0.629	0	4
D13 An urge to hit someone or hurt others	0.07	0.342	0	3
D14 An urge to break or destroy something	0.09	0.422	0	4
D15 Often argue with people	0.18	0.468	0	3
D16 Shouting or throwing things	0.05	0.275	0	3

### Univariate analysis of the patient experience and psychological status

Based on the general condition of patients and the examination services received, factors influencing the medical experience, anxiety, and hostility of patients were analyzed in detail. Based on the characteristics of the data, the independent sample t-test was utilized for the difference analysis of gender, number of children, and number of the elderly family members that they supported, while the Wilcoxon rank sum test was utilized for the difference analysis of marital status, level of education, occupation, monthly income, and MRI examination site. There was a statistically significant difference in the overall medical experiences of patients who underwent MRI examinations in general services (*P* < 0.05), including the notification of time and precautions, explanation of procedures, and the provision of blankets. The score of the anxiety psychological status was significantly higher in patients who were divorced or widowed, had more children, had more elderly family members that they supported, had no occupation, had a monthly income of 3000 or below, and had chest and abdomen MRI examination site (*P* < 0.05). The score of the hostile psychological status was significantly higher in patients who were divorced or widowed, had more elderly family members that they supported, were farmer, and had a monthly income of 3000 or below (*P* < 0.05). The information is presented in Tables 4, 5 and 6.

**Table 4:** Basic information, examination services and patient experience scores of inpatients

N	Categories	Frequency	Percentage (%)	Score of medical experience	Test statistic (T)/rank sum test	*P* value
Gender	Male	119	57.8	56.39 ± 7.159	0.282^a^	0.778
	Female	87	42.2	56.10 ± 7.462		
Marital status	Single	16	7.8	59.00 [53.00,62.00]	1.553^b^	0.460
	Married	187	90.8	56.00 [53.00,59.00]		
	Divorced/widowed	3	1.5	57.00 [55.50,58.50]		
Educational level	Technical secondary school and below	168	81.6	56.00 [53.00,59.00]	2.282^b^	0.319
	Junior college	24	11.7	57.00 [55.00,61.50]		
	Bachelor’s degree or above	14	6.8	58.50 [54.00,60.00]		
Number of children	2 or less	133	64.6	56.12 ± 5.730	-0.351^a^	0.726
	2 or more	73	35.4	56.55 ± 9.50		
Number of the elderly family members supported	2 or less	169	82.0	56.36 ± 6.054	0.257^a^	0.799
	2 or more	37	18.0	55.86 ± 11.397		
Occupation	Farmer	27	13.1	57.00 [51.50, 62.00]	5.722^b^	0.126
	Worker	35	17.0	58.00 [55.00, 61.50]		
	None	44	21.4	56.00 [54.00, 60.00]		
	Others	100	48.5	55.00 [53.00, 59.00]		
Monthly income	RMB 3000 and below	72	35.0	55.50 [52.00, 59.00]	3.088^b^	0.378
	3000–5000	80	38.8	57.00 [54.00, 59.00]		
	5000–8000	49	23.8	55.00 [54.00, 59.00]		
	RMB 8000 and above	5	2.4	58.00 [53.00, 60.00]		
MRI examination site	Head and neck	66	32.0	56.00 [54.00, 59.00]	1.496^b^	0.683
	Chest and abdomen	25	12.1	57.00 [52.00, 64.00]		
	Extremities	6	2.9	54.00 [50.00, 65.00]		
	Others	109	52.9	55.00 [53.00, 59.00]		
Examination service		6.87 ± 1.526			0.217 (Pearson’s coefficient)	0.002

^a^Indicates that differential analysis is performed using the independent samples t-test.

^b^Indicates the use of the Kruskal-Wallis test

**Table 5:** Basic information, examination services and anxiety scores of inpatients

Basic type	Categories	Frequency	Percentage (%)	Score of anxiety	Test statistic (U)/rank sum test	*P* value
Gender	Male	119	57.8	3.00 [2.00, 6.50]	-0.921^a^	0.357
	Female	87	42.2	4.00 [2.00, 7.50]		
Marital status	Single	16	7.8	3.50 [2.50, 9.50]	6.167^b^	**0.046**
	Married	187	90.8	3.00 [2.00, 7.00]		
	Divorced/widowed	3	1.5	12.00 [11.00, 18.00]		
Educational level	Technical secondary school and below	168	81.6	3.00 [2.00, 7.00]	1.281^b^	0.527
	Junior college	24	11.7	4.00 [2.00, 11.50]		
	Bachelor’s degree or above	14	6.8	4.00 [0.00, 10.00]		
Number of children	2 or less	133	64.6	3.00 [2.00, 6.00]	-1.979^a^	**0.048**
	2 or more	73	35.4	5.00 [2.00, 10.00]		
Number of the elderly family members supported	2 or less	169	82.0	3.00 [2.00, 7.00]	-2.181^a^	**0.029**
	2 or more	37	18.0	5.00 [3.00, 10.00]		
Occupation	Farmer	27	13.1	3.00 [3.00, 9.50]	23.906^b^	**0.000**
	Worker	35	17.0	3.00 [1.50, 4.50]		
	None	44	21.4	7.00 [4.00, 13.00]		
	Others	100	48.5	3.00 [2.00, 5.00]		
Monthly income	RMB 3000 and below	72	35.0	5.00 [3.00, 9.50]	13.178^b^	**0.004**
	3000–5000	80	38.8	3.00 [1.50, 5.50]		
	5000–8000	49	23.8	3.00 [2.00, 6.00]		
	RMB 8000 and above	5	2.4	7.00 [1.00, 12.00]		
MRI examination site	Head and neck	66	32.0	4.00 [3.00, 8.00]	9.984^b^	**0.019**
	Chest and abdomen	25	12.1	5.00 [3.00, 10.00]		
	Extremities	6	2.9	3.00 [1.00, 3.00]		
	Others	109	52.9	3.00 [2.00, 6.00]		
Examination service		6.87 ± 1.526			0.062 (Pearson’s coefficient)	0.379

^a^Represents the use of Mann-Whitney U test for differential analysis, and b represents the use of Kruskal-Wallis test

Boldface indicates a statistically significant difference (p < 0.05)

**Table 6:** Basic information, examination services and hostility of inpatients

Basic type	Categories	Frequency	Percentage (%) n	Score of hostility	Test statistic (U)/rank sum test	*P* value
Gender	Male	119	57.8	0.00 [0.00,1.00]	-0.221^a^	0.825
	Female	87	42.2	0.00 [0.00, 1.00]		
Marital status	Single	16	7.8	0.50 [0.00, 2.50]	11.009^b^	**0.004**
	Married	187	90.8	0.00 [0.00, 1.00]		
	Divorced/widowed	3	1.5	6.00 [4.00, 7.00]		
Educational level	Technical secondary school and below	168	81.6	0.00 [0.00, 1.00]	0.781^b^	0.677
	Junior college	24	11.7	0.00 [0.00, 1.00]		
	Bachelor’s degree or above	14	6.8	0.00 [0.00, 1.00]		
Number of children	2 or less	133	64.6	0.00 [0.00, 1.00]	-0.301^a^	0.763
	2 or more	73	35.4	0.00 [0.00, 1.00]		
Number of the elderly family members supported	2 or less	169	82.0	0.00 [0.00, 1.00]	-2.011^a^	**0.044**
	2 or more	37	18.0	0.00 [0.00, 4.00]		
Occupation	Farmer	27	13.1	1.00 [0.00, 3.00]	17.022^b^	**0.001**
	Worker	35	17.0	0.00 [0.00, 0.00]		
	None	44	21.4	1.00 [0.00, 2.50]		
	Others	100	48.5	0.00 [0.00, 1.00]		
Monthly income	RMB 3000 and below	72	35.0	0.00 [0.00, 1.50]	9.647^b^	**0.022**
	3000–5000	80	38.8	0.00 [0.00, 1.00]		
	5000–8000	49	23.8	0.00 [0.00, 1.00]		
	RMB 8000 and above	5	2.4	1.00 [0.00, 3.00]		
MRI examination site	Head and neck	66	32.0	0.00 [0.00, 1.00]	2.821^b^	0.420
	Chest and abdomen	25	12.1	0.00 [0.00, 3.00]		
	Extremities	6	2.9	0.00 [0.00, 1.00]		
	Others	109	52.9	0.00 [0.00, 1.00]		
Examination service		6.87 ± 1.526			0.014(Pearson’s coefficient)	0.842

^a^Represents the use of Mann-Whitney U test for differential analysis, and b represents the use of Kruskal-Wallis test

Boldface indicates a statistically significant difference (p < 0.05)

### Multivariate analysis of patient experience and psychological status

Based on the univariate analysis performed in the previous section, the variables with statistically significant differences were selected as independent variables, and medical experience, anxiety, and hostility of the patients were considered dependent variables. A multiple regression model was established. Model 1: Investigating the influence of medical service (independent variable x) on medical experience (dependent variable y). Model 2: Exploring the effects of marital status (independent variable x_1_), number of children (x_2_), number of the elderly family members that they supported (x_3_), occupation (x_4_), monthly income (x_5_), and MRI examination site (x_6_) on anxiety (dependent variable y). Model 3: Exploring the effects of marital status (independent variable x_1_), number of the elderly family members that they supported (x_2_), occupation (x_3_), and monthly income (x_4_) on hostility (dependent variable y).

Model 1: The univariate regression model was statistically validated (F = 10.066, *P* = 0.002). There was a positive relationship between the examination service experience and the patient’s medical experience, that is, if the examination service improves by 1 unit, the patient’s medical experience satisfaction increases by 1.033 (Table 7).

**Table 7:** Univariate regression analysis of examination service and patient experience

Influencing factors	Unstandardized coefficients		Standardized coefficients	*t* value	*P* value
	Partial regression coefficient	Standard error of coefficients			
Constant	49.174	2.291	—	21.461	0.000
Examination service	1.033	0.326	0.217	3.173	0.002

Model 2: The multiple regression model was statistically validated (F = 7.222, *P* = 0.000). Single (*P* = 0.015), married (*P* = 0.006), unemployed (*P* = 0.000), salary (RMB 5000–8000) (*P* = 0.015), and MRI examination of extremities (*P* = 0.029) were all statistically significant (α = 0.05) predictors of anxiety during medical treatment. Patients with a higher monthly income had a lower anxiety level (coefficient = 2.334), and MRI examinations of the extremities reduced anxiety (coefficient = -4.782). However, patients who were unemployed had a higher anxiety level (coefficient = 3.867) (Table 8).

**Table 8:** Multiple regression analysis of basic information, medical experience and anxiety of inpatients

Model	Unstandardized coefficients		Standardized coefficients	*t*	Significance
	B	Standard error	Beta		
(Constant)	10.826	3.993		2.711	0.007
Experience of the service attitude	0.227	0.124	0.117	1.828	0.069
Single	-7.760	3.177	-0.374	-2.442	0.015
Married	-8.214	2.933	-0.428	-2.801	0.006
Supporting less than 2 elderly family members	-1.660	0.934	-0.115	-1.777	0.077
Unemployed	3.867	0.949	0.285	4.072	0.000
Salary RMB 3000–5000	-1.760	0.896	-0.154	-1.965	0.051
Salary RMB 5000–8000	-2.334	0.951	-0.179	-2.455	0.015
Extremities	-4.782	2.168	-0.145	-2.206	0.029

Model 3: The multiple regression model was statistically verified (F = 6.685, *P* = 0.000). Single (*P* = 0.016), married (*P* = 0.005), unemployed (*P* = 0.010), and supporting two or fewer elderly family members (*P* = 0.006) were all statistically significant (α = 0.05) predictors of hostility during medical treatment. Patients who supported two or fewer elderly family members were less hostile (coefficient = -1.251), while those who were unemployed were more hostile (coefficient = 1.101) (Table 9).

**Table 9:** Multiple regression analysis of basic information, medical experience and hostility of inpatients

Model	Unstandardized coefficients		Standardized coefficients	*t*	Significance
	B	Standard error	Beta		
(Constant)	5.801	1.391		4.171	0.000
Single	-3.549	1.467	-0.383	-2.418	0.016
Married	-3.834	1.359	-0.448	-2.820	0.005
Working	− 0.861	0.468	-0.130	-1.838	0.067
Unemployed	1.101	0.423	0.182	2.602	0.010
Supporting less than 2 elderly family members	-1.251	0.451	-0.194	-2.776	0.006

## Discussion

### Factors affecting the service experience of MRI examination and psychological status of patients

#### Environmental and logistics experience is the most important factor influencing patient experience

The environmental logistics experience was the most influential of the three dimensions of the patient experience survey, with the entry “sound generated by the device during MRI examination” having the greatest impact. It was also related to the examination location and the poor service experience, which included the use of earplugs and the covering of patients with quilts during the examination process. These factors are related to the limitations of the MRI equipment itself. The MRI examination has a longer duration, louder noise, and the patient must maintain a fixed position, all of which are likely to induce a negative mood [26, 27]. However, the service and environment during the examination have a greater impact on patient experience. Provision of accurate information to patients prior to the examination, improved humanistic care and companionship during the process, and provision of accurate information promptly after the examination can have a positive impact on the patient experience [28]. These aspects require further improvement.

#### MRI room services decreased the patient experience

Patients were more satisfied with the service attitude, particularly that of ward nurses, however, the attitude of MRI waiting room staff and the examination procedure received the lowest scores. Presently, a great deal of emphasis has been placed on the medical experience of patients, and ward satisfaction has become an indicator of evaluation and assessment. Hospitals and researchers have been successful in determining how to enhance the patient experience in the ward [29, 30]. However, the services in the MRI examination room are relatively inadequate due to the high patient turnover and the lengthier appointment, examination, and wait times, among other factors. It is relatively easy for patients to be dissatisfied, which in turn affects the mood of the staff, resulting in a lack of patience and an increase in negative emotions. As a result, it is difficult to provide considerate service to patients, resulting in a decline in patient experience and satisfaction.

#### MRI examination has negative effects on the psychological status of patients

The investigation of the psychological status of patients revealed that divorced or widowed status, more children, more elderly family members that they supported, no occupation, lower monthly income, and head/chest MRI examination site, all affected anxiety, while divorced or widowed status, more children, more elderly family members that they supported, with an occupation of farmer, and lower monthly income were significant factors influencing patient hostility. This is due to the fact that medical insurance reimbursement coverage for large equipment is limited and patients are required to bear a significant proportion of the cost. Moreover, the reimbursement ratio of medical insurance for urban residents is greater than that of village residents and uninsured patients, thus urban residents have lower medical expenses. The access to medical insurance for urban residents is reflective of the patients’ relatively stable job and income. China’s medical insurance policy has not yet been implemented nationwide. It is difficult for patients from other regions to seek medical care or to be reimbursed for their medical expenses, leading to an increase in medical costs and a decline in patient experience. Therefore, it is imperative to concentrate on reducing the disparity between various health insurance systems and implementing a national medical insurance policy [31]. 

### Advice for improving the experience of MRI examination and the psychological status of patients

#### Improvement of environmental logistics

Due to the limitations of the MRI equipment itself, examinations take longer and are more noisy. To ensure patients are mentally prepared, they need to be educated on the examination in advance. During the examination, the use of headphones, earplugs, and goggles can be considered, as they are easily accessible, relatively cheap, and effective in isolating equipment noise [32]. To alleviate patient dissatisfaction with the lengthy waiting period, the MRI waiting area could be improved through the addition of more chairs, as well as by the provision of water dispensers and disposable cups, and televisions to broadcast educational videos or television programs about MRI examinations, so that patients are more relaxed, comfortable, diverted, and relieved during the wait [28]. 

#### Improvement in service attitude

Medical staff in MRI examination rooms must enhance their service awareness and implement medical services effectively. A QR code-style real-time experience evaluation and suggestion box can be set up prominently in the MRI examination room, and patients or family members can be requested to conduct on-site real-time evaluation of the services provided by the personnel in the examination room [27, 33]. Through this approach, the MRI examination history of patients and their evaluation results can be routinely reviewed in the backend at any time. Patient satisfaction can be enhanced through rectification based on patient feedback [34, 35]. In the meantime, rich and varied educational and training content could be organized along with guidance on how to behave in a more civilized manner, for medical staff—the evaluation result could be tied to bonus distribution, promotion, and similar incentives to encourage medical staff to improve service quality.

#### Strengthen psychological interventions

The medical staff must improve their communication and education skills, considering varying levels of knowledge and mindsets of patients. Patients can be informed in advance of the reason, purpose, precautions, and costs of an MRI examination in order to alleviate their fears and concerns [36]. During the examination, the patient’s disposition and behavior should be monitored in order to alleviate their anxiety, fear, and other emotions [37]. Following an examination, patients should be promptly informed of the results, and their condition should be communicated in a timely manner in order to avoid unnecessary concerns. Typical examination cases should be analyzed on a daily basis and further enhancements implemented, thereby enhancing the medical staff’s communication abilities [38, 39]. In the meantime, it is advised that medical insurance providers speed up the establishment of a regional medical insurance information sharing platform, which can increase the proportion of medical insurance settlement in different regions, optimize the reimbursement process for medical treatment in different regions, and reduce the burden of personal payment [40]. 

### Limitations

This study was performed in two tertiary hospitals, where the medical environment and services are relatively well-established. Secondly, we focused on inpatients, and the MRI experience of outpatient patients was not investigated. Outpatient patients face issues related to waiting and receiving results, which may differ from the experiences of inpatients. In addition, this study was a single-center, cross-sectional survey study, and the results need further validation through more comprehensive multicenter research. Future studies could explore more detailed research, including subgroup analysis of patients with different conditions and severity levels to understand their demands for medical experiences and the impact on psychological states.

## Conclusion

The MRI examination experience and psychological status of patients are significantly influenced by factors such as environmental and logistical issues, the service attitude and content, the examination site, and economic income. Targeted enhancements can be made to improve the same. Patients can be informed in advance of potential problems, they can be provided noise isolation equipment, the waiting room environment can be enhanced, and patients can be engaged in targeted communication and education to improve their psychological status, and their experience should be evaluated promptly, among other measures. With the advancement of cutting-edge technology, it is possible to improve the MRI examination environment and update the service model of nursing staff in the examination room, which can also be modified based on patient feedback, to enhance patient experience and psychological status. Prior studies on patient experience and its enhancements have centered on hospitalization, the ward environment, and nursing. With the widespread use of large equipment, auxiliary examinations have gradually become an essential part of the patient experience. In the future, hospitals can begin auxiliary evaluations of the equipment environment, service attitude, nursing quality, patient psychological status, and other factors to enhance overall patient satisfaction.

### Electronic supplementary material

Below is the link to the electronic supplementary material.

**Supplementary Material 1**: Questionnaire

## Data Availability

All data generated or analysed during this study are included in this article.
