# Supplementary material for: Investigation and analysis of magnetic resonance imaging experience and psychological status of patients
Source: BMC Psychol. 2024 Mar 1;12:115. doi: 10.1186/s40359-024-01570-7 (PMC10908109; doi:10.1186/s40359-024-01570-7)
Supplement: Supplementary file 1 — Supplementary Material 1: Questionnaire [file 40359_2024_1570_MOESM1_ESM.docx]

**Supplyment**

Supplyment 1 The questionnaire of General information

Dear patient:

Thank you very much for choosing our hospital and filling in our questionnaire during your busy schedule.We sincerely hope you can complete the questionnaire honestly. Your support and participation are very vital that we have your response to this study as a reference for future high-quality service.

A1.Your basic information:

hospital stays (DD/MM/YYYY)

gender age profession

Number of children

Number of elderly dependents currently

A2.Marital status: 1=single; 2=married; 3=divorced; 4=widowed

A3.Education Degree:

1= Secondary school and below ; 2= Junior college;

3= Undergraduate; 4= Master's degree and above

A4. Monthly personal income before taxes:

1$\leq$￥3000; 2=￥3000~; 3=￥5000~;4=￥8000~; 5=￥10000~

A5. Examination time of MRI (DD/MM/YYYY)

A6.Site of MRI

Supplyment 2 The service's content when MRI examine^[1-2]^

B1.Was there someone who can take you from the ward to the MRI room?

1=Yes; 2=No

B2.Before the examination,whether the ward nurse informed you of the examination time ?

1=Yes; 2=No

B3.Whether the ward nurse educated you about the precautions of the examination?

1=Yes; 2=No

B4.Whether the MRI room nurse educated you about the precautions of the examination?

1=Yes; 2=No

B5.Before the examination,Whether the staff asked for your name?

1=Yes; 2=No

B6.Before the examination, Whether the staff asked for your examination site?

1=Yes; 2=No

B7.Were you instructed on how to notify the staff if something goes wrong during the inspection?

1=Yes; 2=No

B8.Whether to plug your ears during the examination?

1=Yes; 2=No

B9.Whether to tuck you in during the examination?

1=Yes; 2=No

Supplyment 3 The questionnaire of MRI service Experience^[1-2]^

Part One: The expertise with environmental logistics

C1.Inpatient ward is quiet:

1= very dissatisfied; 2= less satisfied; 3 = general; 4 = relatively satisfied; 5 = Very satisfied

C2.The MRI waiting area is quiet:

1= very dissatisfied; 2= less satisfied; 3 = general; 4 = relatively satisfied; 5 = Very satisfied

C3.The sound produced by the device during an MRI examination:

1= very dissatisfied; 2= less satisfied; 3 = general; 4 = relatively satisfied; 5 = Very satisfied

C4.The temperature of MRI to check in the waiting area:

1= very dissatisfied; 2= less satisfied; 3 = general; 4 = relatively satisfied; 5 = Very satisfied

C5.The temperature of MRI examination room:

1= very dissatisfied; 2= less satisfied; 3 = general; 4 = relatively satisfied; 5 = Very satisfied

Part Two: The experience with disease transmission

C6.The content of ward mission:

1= very dissatisfied; 2= less satisfied; 3 = general; 4 = relatively satisfied; 5 = Very satisfied

C7.The content of ward nurse mission:

1= very dissatisfied; 2= less satisfied; 3 = general; 4 = relatively satisfied; 5 = Very satisfied

C8.Language used in MRI room missions:

1= very dissatisfied; 2= less satisfied; 3 = general; 4 = relatively satisfied; 5 = Very satisfied

C9.The explanation given by the staff in response to your queries:

1= very dissatisfied; 2= less satisfied; 3 = general; 4 = relatively satisfied; 5 = Very satisfied

C10.Check the process of personal privacy protection：

1= very dissatisfied; 2= less satisfied; 3 = general; 4 = relatively satisfied; 5 = Very satisfied

Part Three:The experience Of Service attitude

C11.The attitude of Ward nurse service:

1= very dissatisfied; 2= less satisfied; 3 = general; 4 = relatively satisfied; 5 = Very satisfied

C12.The service attitude of the staff in the MRI waiting area:

1= very dissatisfied; 2= less satisfied; 3 = general; 4 = relatively satisfied; 5 = Very satisfied

C13.The service attitude of technicians during MRI examinationhe:

1= very dissatisfied; 2= less satisfied; 3 = general; 4 = relatively satisfied; 5 = Very satisfied

C14.The overall satisfaction of the whole inspection:

1= very dissatisfied; 2= less satisfied; 3 = general; 4 = relatively satisfied; 5 = Very satisfied

Supplyment 4 The scale of Symptom self-assessment^[3]^

Part one: Anxiety

D1.Neurotic, not in the heart：

1. no ② mild ③ moderate ④quit heavy ⑤ serious

D2.Tremble:

①no ② mild ③ moderate ④quit heavy ⑤ serious

D3.Suddenly feel scared for no reason:

①no ② mild ③ moderate ④quit heavy ⑤ serious

D4.Feel scared:

①no ② mild ③ moderate ④quit heavy ⑤ serious

D5.The heart beat violently:

①no ② mild ③ moderate ④quit heavy ⑤ serious

D6.Feel nervous or get nervous easily:

①no ② mild ③ moderate ④quit heavy ⑤ serious

D7.Bouts of fear or panic:

①no ② mild ③ moderate ④quit heavy ⑤ serious

D8. Feel restless and uneasy:

①no ② mild ③ moderate ④quit heavy ⑤ serious

D9.Feel strange or unreal for something that familiar:

①no ② mild ③ moderate ④quit heavy ⑤ serious

D10.Feel the need to get things done quickly:

①no ② mild ③ moderate ④quit heavy ⑤ serious

Part Two: Hostility

D11.Easily upset and excited:

①no ② mild ③ moderate ④quit heavy ⑤ serious

D12.Lose your temper uncontrollably:

①no ② mild ③ moderate ④quit heavy ⑤ serious

D13.The urge to hit or hurt others:

①no ② mild ③ moderate ④quit heavy ⑤ serious

D14.An urge to break or destroy things:

①no ② mild ③ moderate ④quit heavy ⑤ serious

D15.Argue with people often:

①no ② mild ③ moderate ④quit heavy ⑤ serious

D16.Yelling or throwing things:

①no ② mild ③ moderate ④quit heavy ⑤ serious

References

1. Chang Yubo, Chen Jiajun,Jia Xiaochan, et al. Screening of inpatient experience scale items.Journal of Zhengzhou University: Medical Edition,2013(3):4.
2. Tian Changjun. Evaluation of medical service quality based on patient experience.Huazhong University of Science and Technology, 2014.
3. Wang Zhengyu. Symptom checklist 90 (SCL-90). Shanghai Archives of Psychiatry,1984(2).
